# Complication of Hepatitis A Infection: Case Report of Acute Inflammatory Demyelinating Polyneuropathy

**DOI:** 10.5811/cpcem.2020.9.48827

**Published:** 2020-11-25

**Authors:** Daniel Laursen, Jeffrey Krug, Robert Wolford

**Affiliations:** University of Illinois College of Medicine – Peoria, Department of Emergency Medicine, Peoria, Illinois. OSF Saint Francis Medical Center, Department of Emergency Medicine, Peoria, Illinois

**Keywords:** Hepatitis A, acute inflammatory demyelinating polyneuropathy, Guillain-Barré syndrome

## Abstract

**Introduction:**

Acute inflammatory demyelinating polyneuropathy (AIDP) is characterized by progressive, mild sensory symptoms and progressive areflexic weakness. It typically follows a gastrointestinal or respiratory infection but has rarely been described after acute viral hepatitis.

**Case Report:**

This is the case of a 59-year-old male who presented to the emergency department after acutely developing progressive neurologic symptoms following a hospitalization for acute hepatitis A. Cerebrospinal fluid analysis revealed albuminocytologic dissociation, and cervical spine magnetic resonance imaging revealed nerve root enhancement.

**Discussion:**

The patient was diagnosed with AIDP, which is the most common subtype of Guillain-Barré syndrome in the United States and Europe. There have been few previously reported cases of AIDP following acute hepatitis A infection.

## INTRODUCTION

Approximately 6700 cases of hepatitis due to hepatitis A virus (HAV) occurred in the United States (US) in 2017. Typically, hepatitis due to HAV is self-limiting, resolves spontaneously, requires only supportive care, and has a very low mortality rate. Rarely, there are extrahepatic complications of the disease. We report one such complication.

Acute inflammatory demyelinating polyneuropathy (AIDP), the most common variant of Guillain-Barré syndrome (GBS), is an autoimmune condition classically characterized by loss of reflexes, ascending weakness, cranial nerve involvement, and subtle sensory changes. In the US, 3000–6000 persons are diagnosed annually. Symptoms typically follow an upper respiratory or gastrointestinal infection in the preceding 1–6 weeks.^1^ Admission to an intensive care setting is typical, as these patients can develop precipitous ascending weakness, respiratory failure (20% of AIDP patients), and autonomic dysfunction.^1^ Early recognition of AIDP and appropriate disposition and treatment are key to improve outcomes and avoid complications. Thus, it is important for the emergency clinician to develop a broad list of differential diagnoses when patients present with neurologic complaints and physical findings. This is a case of AIDP that followed an acute hepatitis A infection.

## CASE REPORT

A 59-year-old male presented to the emergency department (ED) with a two-day history of weakness, numbness, and tingling bilaterally in the hands, feet, and legs. Five days earlier he had been discharged from the hospital after a three-day admission due to acute HAV hepatitis. Hepatitis A was diagnosed at that time with a serologic acute hepatitis panel that revealed hepatitis A immunoglobulin M antibody. He denied vision changes, difficulty with speech or swallowing, dizziness, difficulty breathing, headaches, or bowel and bladder complaints. Gait was wide-based and mildly ataxic. A non-contrast head computed tomographic (CT) was obtained and was normal.

Lumbar puncture was performed by the neurology service. The cerebrospinal fluid (CSF) protein was found to be elevated at 68.8 milligrams per deciliter (mg/dL) (reference range 12–60 mg/dL). Two nucleated cells and three red blood cells were found in tube one from the lumbar puncture. Lyme antibody, West Nile virus antibody, Gram stain, and CSF cultures were all found to be negative. Lead levels were below reference range and immunoglobulin A levels within normal limits. The patient was empirically started on intravenous immunoglobulin, (IVIG), for treatment of AIDP. During his hospital admission, he developed objective weakness in bilateral upper and lower extremities, ascending loss of reflexes, and decreased vibratory sense and a positive Babinski sign in the left lower extremity (Tables 1, 2). Due to this observed asymmetry, magnetic resonance imaging (MRI) was performed to evaluate for myelitis. MRI revealed enhancement of the first through seventh cervical spine nerve roots, which may be seen with AIDP (Image), and no evidence of myelitis. Prior to discharge, the patient also developed decreased vibratory sense in his right lower extremity without a positive Babinski sign. He was discharged after receiving five days of IVIG. He never developed respiratory symptoms, and his negative inspiratory force testing remained within normal limits throughout the hospital stay. Four days after discharge, when the patient was seen in the neurology clinic, he required the use of a wheelchair for mobility. However, approximately two weeks later he no longer used the wheelchair, as his strength was returning, and he was working with physical therapy. At follow-up approximately five months post-discharge, the patient’s gait had nearly returned to baseline. He did endorse mild continued fingertip paresthesias, but those symptoms were slowly improving.

## DISCUSSION

Acute inflammatory demyelinating polyneuropathy has been associated with a variety of preceding etiologies, including viral and bacterial infections, severe acute respiratory syndrome coronavirus 2 infection,^9^ vaccinations, and malignancy.^1^ *Campylobacter jejuni*, human immunodeficiency virus, and Epstein-Barr virus are commonly identified illnesses that precipitate AIDP. Hepatitis is rarely reported as a preceding infection. Hepatitis A, similar to other infections preceding AIDP, is thought to cause a dysregulated immune response against myelin, a result of cross-reactivity and molecular mimicry.^7^ There have also been reported cases of hepatitis A preceding other variants of GBS, including acute motor axonal neuropathy, in which the patients suffer from isolated ascending motor symptoms.^3^,^7^,^8^

**CPC-EM Capsule**

What do we already know about this clinical entity?

Acute inflammatory demyelinating polyneuropathy (AIDP), an autoimmune disease with weakness and sensory changes, commonly follows a variety of infections.

What makes this presentation of disease reportable?

Although associated with respiratory and gastrointestinal infections, AIDP is not commonly reported to follow hepatitis due to hepatitis A virus.

What is the major learning point?

The differential diagnosis of patients with a recent history of hepatitis A infection and complaints of weakness and/or sensory changes should include AIDP.

How might this improve emergency medicine practice?

Early recognition and management of AIDP is essential to guide appropriate emergency department disposition, avoid complications, and improve patient outcomes.

The neurology service was initially skeptical of the AIDP diagnosis, given recent acute hepatitis A infection and few previously reported cases of antecedent hepatitis A infections. Although our patient did not have nerve conduction testing, it is not essential in making the AIDP diagnosis, which can be made based on the clinical course of the illness and laboratory findings. Our patient’s clinical presentation and laboratory findings were consistent with AIDP and met level 2 of diagnostic certainty by Brighton criteria.^4^ The patient had bilateral limb weakness, along with decreased and absent deep tendon reflexes in the weakened limbs. He also experienced a monophasic pattern of symptoms that – by chart review – nadired within 28 days of onset. There was an absence of better alternative diagnosis to explain the patient’s progressive ascending symptoms.

The total CSF white blood cell count of less than 50 and elevated CSF protein, known as albuminocytologic dissociation, was also consistent with AIDP. MRI is usually not used in the diagnosis of AIDP, but rather to exclude other diagnoses such as transverse myelitis and acute flaccid myelitis. This patient did have MRI findings of spinal root enhancement, which has been previously reported in GBS.^2^ During his hospital course, the patient had paroxysms of tachycardia and hypertension, likely related to the dysautonomia, which is a common clinical feature associated with GBS.^6^

The patient began showing signs of improvement approximately four weeks after the initial onset of symptoms, which is also consistent with the majority of cases of AIDP.^5^ Of note, at the time of the patient’s initial admission for treatment of acute HAV hepatitis, he had been taking amoxicillin-clavulanate for five days to treat sinusitis. After discussion with the patient, his symptoms did not seem to be consistent with sinusitis. Instead, the generalized malaise, joint pains, chills, and nausea he had complained of and which had led to the antibiotic prescription, were likely related to his HAV infection.

## CONCLUSION

This patient had a convincing diagnosis of acute inflammatory demyelinating polyneuropathy, even in the absence of electrophysiologic testing. Many different antecedent illnesses may lead to AIDP. In this case, it appears viral hepatitis A was the cause. Although there are few reported cases of AIDP following acute viral hepatitis A infection, it is worthy of consideration when evaluating ED patients with neurologic complaints.

## Figures and Tables

**Image:**
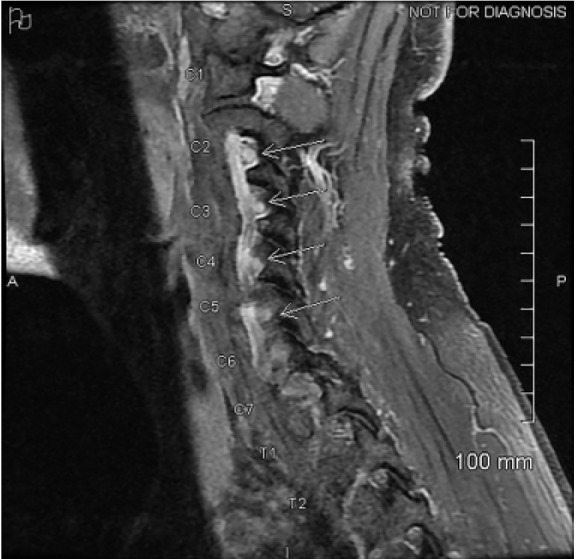
Magnetic resonance imaging of the cervical spine with and without contrast revealed enhancement of the cervical spine nerve roots (arrows), which may be seen in acute inflammatory demyelinating polyneuropathy.

**Table 1:** Cranial nerve and sensory examination findings during hospital stay of patient with 2-day history of weakness and bilateral numbness and recent history of acute hepatitis A infection.

	Cranial nerves	Sensory exam
ED presentation	Fully intact	Fully intact
Day 2	Fully intact	Decreased VS left LE
Day 3	Fully intact	Decreased VS left LE
Day 4	Fully intact	Decreased VS b/l LE
Day 5	Fully intact	Decreased VS b/l LE

*ED,* emergency department; *VS*, vibratory sensation; *LE*, lower extremity; *b/l*, bilateral.

**Table 2:** Strength and deep tendon reflex examination findings during hospital stay.

	UE Strength	LE Strength	UE DTR	LE DTR
ED presentation	Fully intact	Fully intact	Brachioradialis: 1/4 b/l	Patellar: trace b/l Ankle jerk: 0/4 b/l
Day 2	Fully intact	Hip Flexors: 4/5 b/l Quadriceps: 4/5 b/l Hamstrings: 4/5 b/l	Triceps: 1/4 b/l	Patellar: 0/4 b/l Ankle jerk: 0/4 b/l
Day 3	Biceps: 4/5 right Triceps: 4/5 right	Hip Flexors: 4/5 b/l Quadriceps: 4/5 b/l Hamstrings: 4/5 b/l	Biceps: 1/4 b/l Brachioradialis: 1/4 b/l Triceps: 0/4 b/l	Patellar: 0/4 b/l Ankle jerk: 0/4 b/l Upgoing left Babinski Absent right plantar response
Day 4	Biceps: 4/5 right Triceps: 4/5 right Grip: 4/5 b/l	Hip Flexors: 4/5 b/l Quadriceps: 4/5 b/l Hamstrings: 4/5 b/l	Biceps: 1/4 b/l Brachioradialis: 0/4 b/l Triceps: 0/4 b/l	Patellar: 0/4 b/l Ankle jerk: 0/4 b/l Upgoing left Babinski Absent right plantar response
Day 5	Biceps: 4/5 right Triceps: 4/5 right Grip: 4/5 b/l	Hip Flexors: 4/5 b/l Quadriceps: 4/5 b/l Hamstrings: 4/5 b/l	Biceps: 1/4 b/l Brachioradialis: 0/4 b/l Triceps: 0/4 b/l	Patellar: 0/4 b/l Ankle jerk: 0/4 b/l

*ED,* emergency department; *UE*, upper extremity; *LE*, lower extremity; *DTR*, deep tendon reflex; *b/l*, bilateral.
